# First Reported Case of Bronchoperitoneal Fistula From Bladder Cancer Metastasis Treated With the Novel Utilisation of an Endobronchial Valve

**DOI:** 10.7759/cureus.71025

**Published:** 2024-10-07

**Authors:** Matthew Fitzpatrick, Gabrielle R Yankelevich, Jennifer Drerup, Robert L Grubb

**Affiliations:** 1 Urology, Edward Via College of Osteopathic Medicine, Spartanburg, USA; 2 Urology, Medical University of South Carolina, Charleston, USA

**Keywords:** bladder cancer, bronchoperitoneal fistula, endobronchial valve, interventional pulmonology, metastasis, pneumoperitoneum

## Abstract

Bronchoperitoneal (BP) fistulas are exceedingly rare and complicated conditions. Given the rarity of this diagnosis, with reports limited to case reports or case series, there is limited literature regarding treatment, which ranges from conservative to surgical management. A search for 'bronchoperitoneal fistula from bladder cancer' on PubMed and Google Scholar revealed no reported studies. Therefore, to our knowledge, this is the first case of BP fistula caused by metastatic bladder cancer. Moreover, to our knowledge, we present the second instance in the literature of utilizing an endobronchial valve to treat a BP fistula. Ultimately, the procedure was successful and allowed for improved quality of life.

## Introduction

Bronchoperitoneal (BP) fistulas are exceedingly rare and complicated conditions. These pathologic connections have been sparsely recorded in the literature but have been attributed to thoracoabdominal trauma, pulmonary abscess, radiation, malignancy, and biliary tree operations [1-6]. They can be life-threatening, as patients are at high risk for pulmonary decompensation and infections. 

Incidence from malignancy has been attributed to colon or biliary cancer [1-2,6]. To our knowledge, we report the first case stemming from a bladder cancer metastatic lesion. There were no reports or studies for the search term 'bronchoperitoneal fistula from bladder cancer' on PubMed and Google Scholar. This case report explores this rare entity in a 77-year-old male whose metastatic bladder cancer ultimately led to a BP fistula. Moreover, we present the novel placement of an endobronchial valve as a treatment for this rare condition. 

## Case presentation

The patient is a 77-year-old male with a past medical history of type II diabetes mellitus, remote head/neck squamous cell carcinoma treated with radiation (XRT) followed by salvage laryngectomy in 2017 without recurrence, and bladder cancer with metastasis. He was initially diagnosed with muscle-invasive bladder cancer (MIBC) in April 2022 after presenting for a microscopic hematuria evaluation. He was found to have a left-sided bladder tumor and was taken for a transurethral resection with pathology demonstrating high-grade urothelial carcinoma with detrusor muscle invasion. Staging imaging was without metastasis, so he was initiated on neoadjuvant cisplatin and gemcitabine before the planned cystectomy.

Unfortunately, in September 2022, he developed local progression involving the left bladder wall and ureter after only four cycles of chemotherapy, which required a left-sided percutaneous nephrostomy tube (PCNT) to relieve the obstruction. Given he was no longer a cystectomy candidate due to progression, his regimen was changed to pembrolizumab and enfortumab vedotin at this time. In 2023, he developed malignant obstruction of the right side due to further progression of the disease and required placement of a right PCNT. At this time, he was also noted to have vertebral lesions and metastatic deposits on the right hemidiaphragm, lungs (right upper lobe, right middle lobe, and right lower lobe involving the diaphragm), and liver. He went on to undergo palliative pelvic XRT in April 2023 for ongoing pelvic pain and was transitioned from pembrolizumab to sacituzumab govitecan due to hepatic metastases.

**Figure 1:**
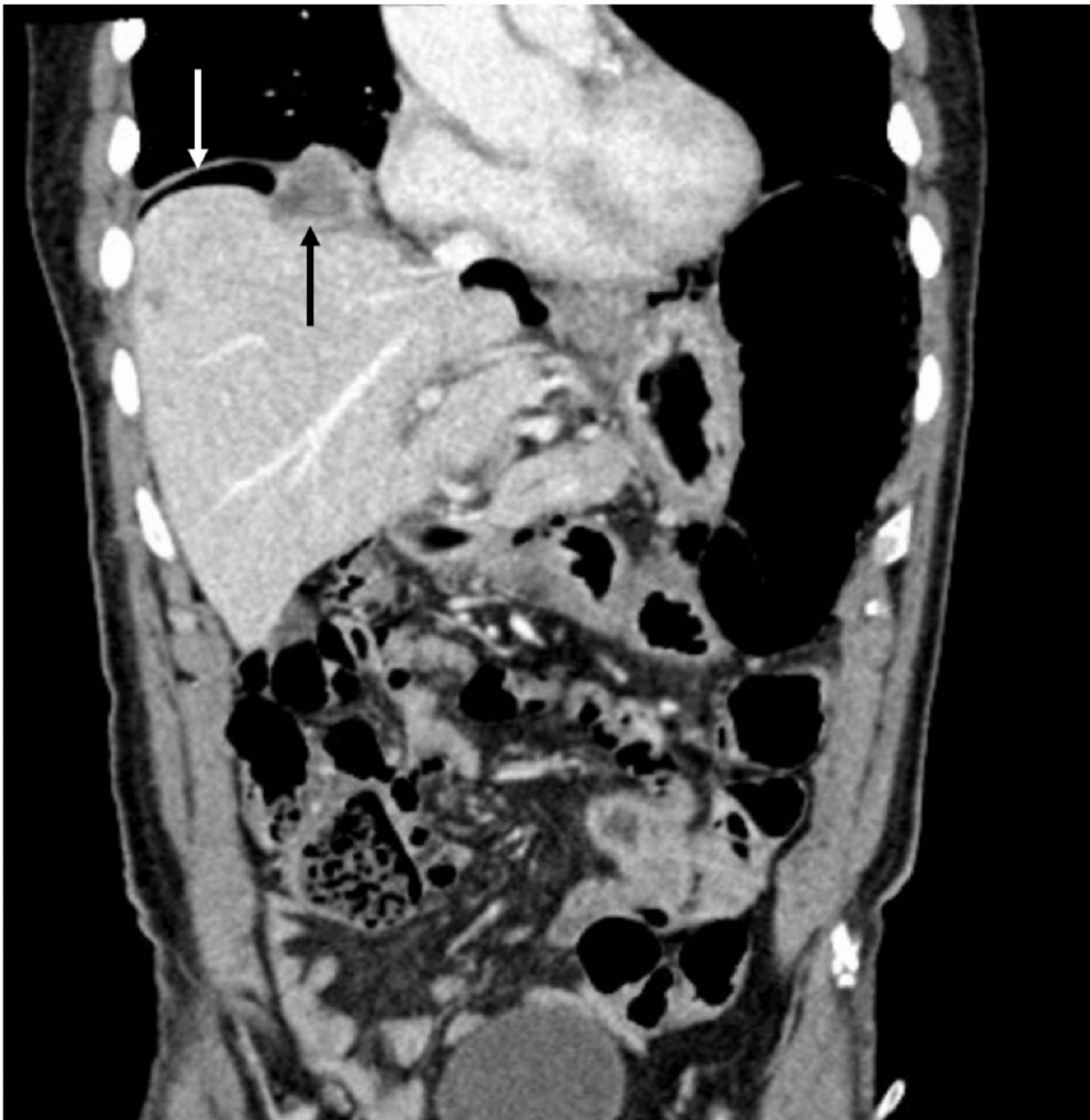
Coronal view of CT abdomen pelvis showing right-sided diaphragmatic lesion (black arrow) with pneumoperitoneum (white arrow)

**Figure 2:**
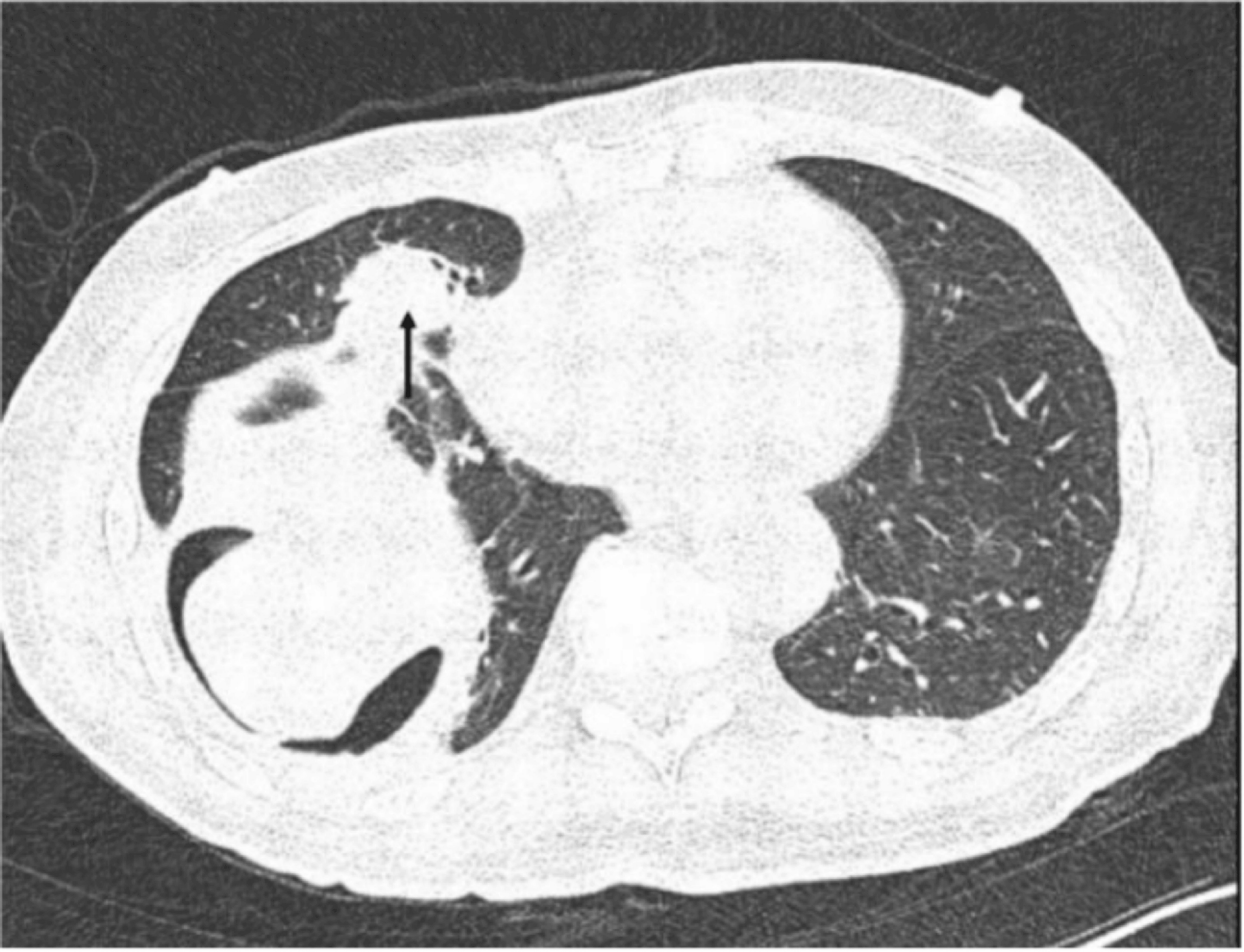
Axial view of CT chest (lung window) with right-sided diaphragmatic lesion (black arrow)

In March 2024, he remained on sacituzumab govitecan therapy and presented to the outpatient urology clinic with a complaint of abdominal pain. He was hemodynamically stable and saturating at 98% on room air. Same-day CT imaging demonstrated the progression of known vertebral body lesions and right hemidiaphragm lesions, with new findings of small volume upper abdominal pneumoperitoneum (Figure 1). The patient was admitted from the clinic to the hospital for further workup. In the setting of pneumoperitoneum, there was high suspicion of the development of a BP fistula from the diaphragmatic metastasis, so a dedicated CT chest was obtained, which confirmed the diagnosis (Figure 2). There was no pneumothorax as the bronchial tissue was involved, not the pleural tissue.

We consulted the cardiothoracic and general surgery teams to consider a wedge lung resection and/or diaphragmatic resection, but they deemed him a poor surgical candidate due to his metastatic disease and poor functional status. The pulmonology team was consulted for consideration of palliative endobronchial valve placement to prevent pulmonary decompensation and discomfort. He was taken for bronchoscopy and valve insertion at the right middle lobe and the right lower lobe on hospital day three. He was discharged home on hospital day seven.

**Figure 3:**
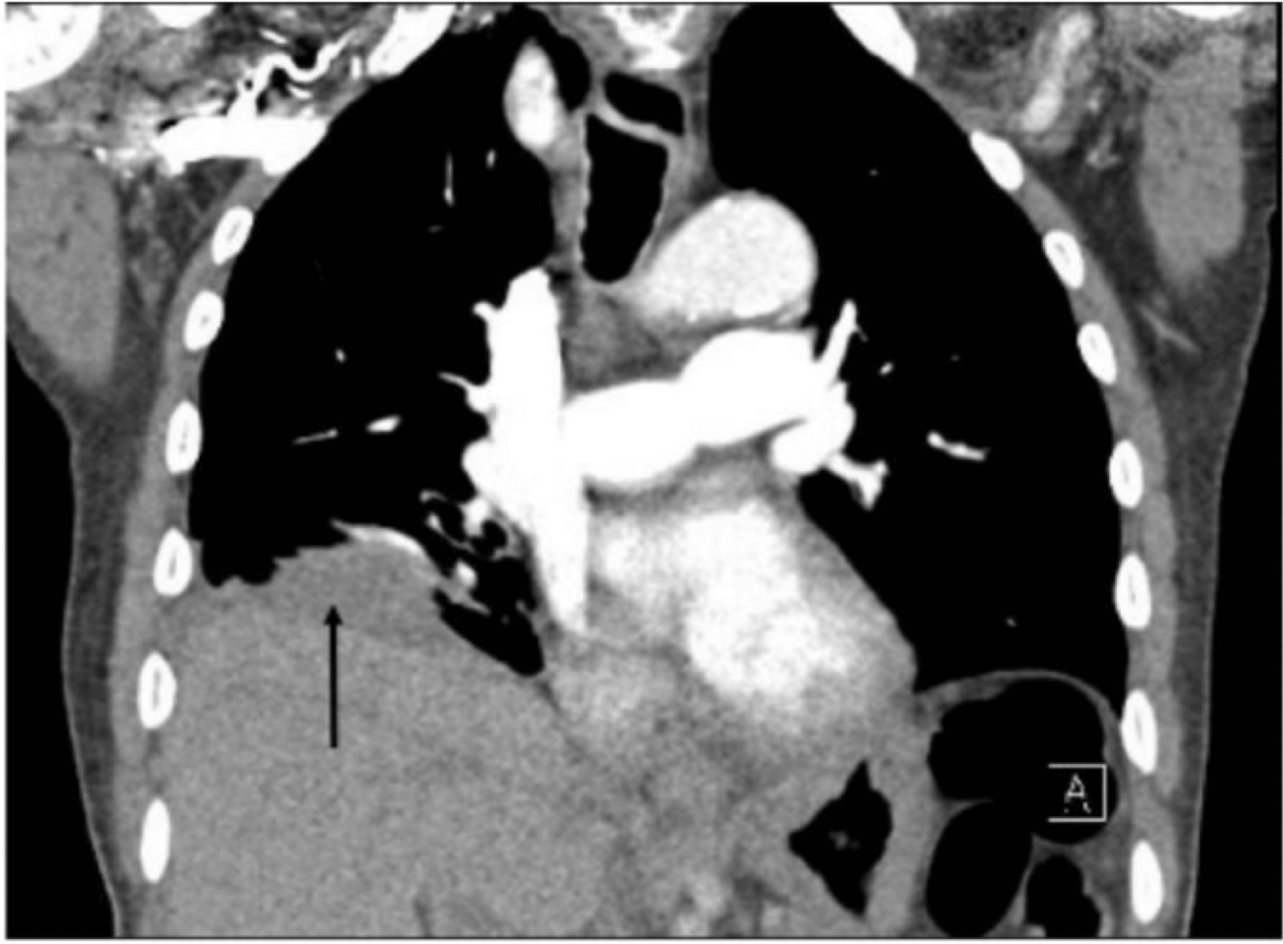
Coronal view of CT abdomen pelvis showing right-sided diaphragmatic lesion (black arrow) without evidence of pneumoperitoneum

To date, he has completed a course of palliative radiation to the L1 lytic lesion due to ongoing pain. A CT chest pulmonary embolism protocol was obtained in May 2024 for tachypnea with a stable size of diaphragmatic nodule but without evidence of pneumoperitoneum and no evidence of pulmonary embolism (Figure 3). After this episode, he elected to pursue hospice care.

## Discussion

Bronchoperitoneal fistulas are rare diagnoses that can arise from malignancy, abscess, trauma, inflammatory processes, or biliary tree operations [1-6]. A search on PubMed and Google Scholar showed no reports of fistulas arising from metastasis. These fistulas are incredibly dangerous for patients, increasing their likelihood of infection, tracheostomy, and acute respiratory distress syndrome (ARDS) [1,3]. One primary goal of treatment is to reduce the amount of air flowing close to the fistula. Specifically, when considering the possibility of ARDS, it has been suggested that the forced positive end-expiratory pressure (PEEP) should be lower than normal, while the fraction of inspired oxygen (FiO2) should be higher than normal, along with subdiaphragmatic drainage [4]. The diagnosis of BP fistula is usually confirmed by a CT scan. In the setting of colobronchial fistulas, endoscopy has also been described for diagnosis [6].

Given the rarity of this diagnosis, there is limited literature on the treatment. The existing literature has suggested treatments ranging from utilizing conservative management to surgical management. Few cases delineate surgical intervention to excise the fistula tract and repair the diaphragmatic defect [1,7]. There are also a few reports in the literature that employed drain placement, such as the one by Kumar et al. They reported a BP fistula caused by a *Klebsiella pneumoniae* infection, which was treated conservatively by percutaneous drainage and antibiotics [3].

In patients who are not surgical candidates, there has been one published case report by Zoumot et al. in which interventional pulmonology placed an endobronchial valve to limit airflow [8]. The patient had a large diaphragmatic defect with BP fistula after undergoing multiple bowel surgeries for anastomotic leak after Roux-en-Y esophagojejunostomy surgery. The patient was evaluated by general surgery and thoracic surgery for consideration of closure of the diaphragm or thoracoscopic intervention, but they felt the likelihood of successful closure was low and outweighed by a high risk of morbidity and mortality to the patient [8]. The interventional pulmonologists placed a Zephyr endobronchial valve with immediate improvement in tidal volumes and oxygen saturations [8]. The CT imaging three days later showed a decreased volume of pneumoperitoneum, and eventually, the patient was able to undergo partial esophagectomy and left pulmonary decortication [8].

To our knowledge, we present the second case in the literature of utilizing an endobronchial valve for a BP fistula. There have been no suggestions for follow-up in the literature, but we would suggest interval imaging with a CT scan based on patient symptoms, clinical presentation, and goals of treatment.

## Conclusions

Ultimately, BP fistula is a rare diagnosis, and clinicians should be able to recognize and diagnose this disease process. A BP fistula rarely occurs from malignant etiology, but we present the first reported case of metastatic bladder cancer leading to a BP fistula. Therefore, it should be considered in the differential for metastatic patients with respiratory symptoms. Lastly, we offer a solution for poor surgical candidates by placing a palliative endobronchial valve.
